# The Effect of Thermal Processing on the Microstructure and Properties of a Novel Nickel-Based Powder Metallurgy Superalloy

**DOI:** 10.3390/ma18051018

**Published:** 2025-02-25

**Authors:** Jiangying Xiong, Chao Yin, Anping Long, Junyi Cheng, Ganjiang Feng, Jianzheng Guo

**Affiliations:** 1 State Key Laboratory of Powder Metallurgy, Central South University, Changsha 410083, China; xiong1988@csu.edu.cn (J.X.); 193302073@csu.edu.cn (A.L.); 223302084@csu.edu.cn (J.C.); fenggj@wedge.com.cn (G.F.); 2 Wedge Central South Research Institute Co., Ltd., Shenzhen 518045, China; 18573792948@163.com

**Keywords:** powder metallurgy disk alloy, thermal processing, microstructure, mechanical property

## Abstract

A novel nickel-based powder metallurgy superalloy was processed using two different thermal–mechanical processes, including hot isostatic pressed (As-HIP) and hipped + hot extruded + isothermally-forged (IF) heat treatments following two processed alloys, designated as As-HIP-HT and IF-HT. The objective of this study is to investigate the microstructure and mechanical property evolution in a nickel-based powder disk alloy fabricated by two processes. The findings revealed that both As-HIP and IF alloys underwent substantial recrystallization, with grains in the IF alloy being finer. Notable Prior Particle Boundaries (PPBs) were identified in the As-HIP samples. The IF-HT alloy exhibited a larger grain size due to a greater amount of stored energy. Significant differences in the secondary γ′ precipitates were observed between the two processes. More uniform substructures in the IF-HT alloy led to a higher density of finer γ′ precipitates. At temperatures of 704 °C and 760 °C, the As-HIP-HT alloy displayed a higher yield strength, but its plasticity significantly declined as temperature increased, while the IF-HT alloy showed a relatively stable plasticity. The presence of PPBs in the As-HIP-HT alloy minimally affected the alloy’s strength but reduced its plasticity. The creep property of the two processes was compared at 800 °C/330 MPa; the IF-HT alloy demonstrated lower creep rates and a longer creep life, which was attributed to its finer γ′ precipitates. Dominant creep deformation mechanisms in the As-HIP-HT alloy included Orowan dislocation loops and deformation twinning, while the primary mechanisms in the IF-HT alloy involved dislocation cutting through γ′ precipitates, dislocation slip, and micro-twins. These findings support the use of isostatic pressing + hot extrusion+ isothermally-forging process for critical high-temperature components.

## 1. Introduction

With the evolving demands of the next generation of aircraft engines, critical hot-end components, such as high-pressure turbine disks, must function under elevated temperatures and complex stress conditions for extended durations. As a result, a new type of powder superalloy has been developed to enhance temperature tolerance [1]. Those novel powder superalloys possess high strength, excellent damage tolerance, durability, and the capability to operate at a temperature of 750 °C for prolonged periods and reach up to 800 °C for short durations. The new powder disk alloys developed abroad in the past twenty years include René104 (ME3) [2], RR1000 [3,4], and LSHR [5], which have been widely utilized in jet turbine engines. In the meantime, China has developed several similar types of powder disk alloys, such as FGH98 [6], FGH99, and FGH101. Most of these alloys are still in the development and trial stage, with limited information available. FGH4113A represents a newly developed powder superalloy that maintains thermal strength while enhancing structural stability by tailoring the Ta and Nb content. By achieving a high Co content, minimizing the precipitation of the TCP phase, and optimizing the precipitation behavior of γ′, the alloy attains superior high-temperature strength [7,8,9,10].

The preparation of nickel-based powder superalloys typically involves hot isostatic pressing (HIP) followed by heat treatment (HT) to produce components like baffles, ring parts, and shaft parts. Both the Russian EP741NP disk and the recently developed BB series turbine disks adhere to this processing route [11,12]. For turbine disks, which are critical rotor components with stringent performance requirements, the preparation processes can include either HIP + isothermally-forging (IF) + HT or HIP + hot extrusion (HEX) + IF + HT processes [2,13]. Different preparation methods significantly affect the microstructure and mechanical properties of powder alloys.

As-HIP and HIP + HEX + IF are prominent methods for producing powder superalloys and have received considerable attention. Research on HIP encompasses the effects of HIP temperature and the HT scheme on the microstructure and properties of alloys [14,15,16]. May et al. [16] investigated the microstructure and tensile properties of an RR1000 alloy in the As-HIP state, with varying power particle sizes, following sub-solvus and sup-solvus solution heat treatments. They found that fine-grained powder alloys with high concentration of PPBs, which exerts a pinning effect on grain boundaries, effectively hinder grain growth. The tensile strength, yield strength, and elongation of the As-HIP sample are comparable to the disk processed through HEX and IF for RR1000. Rao et al. [17] discovered that, after undergoing solution treatment followed by two-stage aging, the As-HIP state U720Li alloy exhibited finer grains and a large amount of rectangular secondary γ′ precipitates. Additionally, MC carbide films were formed at the grain boundaries. Compared to the specifications for forging alloys intended for high-strength applications, the As-HIP state U720Li alloy, despite having slightly lower plasticity, fulfilled the requirements for tensile strength and yield strength at both room temperature and 650 °C, as well as creep life at 680 °C/830 MPa and 730 °C/530 MPa, far exceeded the technical requirements. It is evident that both the As-HIP RR1000 and U720Li alloys exhibit promising potential to manufacture near-net-shaped components for aerospace applications.

There are numerous reports on the microstructure and properties of nickel-based powder disk alloys subjected to the HIP + HEX + IF process. Gabb et al. [2] performed a comprehensive examination of the microstructure and performance of powder turbine disk alloy ME3 and demonstrated that this process satisfied the design performance requirements for turbine disks in engines operating at temperatures between 704 °C and 732 °C. Ning et al. [18] obtained ultrafine-grained powder superalloy billets by undergoing repeated multi-directional deformation processes. The repeated multi-directional deformation process accumulated a significant amount of strain energy, triggering extensive recrystallizations in the alloy. Compared to the HIP-only process, the forged alloys exhibited a higher grain boundary cleanliness, fewer defects, and fine tertiary γ′ precipitates within the matrix, which was beneficial for the alloy’s high-temperature mechanical properties. Furthermore, the enhanced coordinated deformation ability between grains and grain boundaries contributes to the alloy’s high plasticity. Peng et al. [19] investigated the creep behavior of FGH96 alloy. When the temperature is in the range of 650–750 °C and the stress is in the range of 690–810 MPa, the creep deformation of FGH96 alloy is primarily dominated by dislocation slip.

This study presents the preparation of the novel nickel-based powder superalloy FGH4113A by As-HIP and HIP + HEX + IF. The objective is to conduct a thorough comparison of microstructure and mechanical properties after different processes and subsequent heat treatments. The goal is to analyze microstructural evolution, mechanical behavior, creep performance, and deformation mechanisms, ultimately providing more information for the processes and materials selection of powder disk alloys in diverse application scenarios.

## 2. Materials and Methods

### 2.1. Materials

FGH4113A was used in the current study with the nominal chemical composition (mass fraction, wt.%) of Co 19.0, Cr 13.0, Mo 4.0, W 4.0, Al 3.0, Ti 3.7, Nb 1.2, Ta 1.0, Hf 0.2, C 0.05, Zr 0.05, and B 0.03, with the balance being Ni. The alloy powder was prepared via argon gas atomization using a VIGA-100kg vacuum induction argon atomization machine (ZHMT, Beijing, China), resulting in powder particle sizes of ≤53 µm after screening. The powder was loaded into a stainless steel can, degassed, and sealed in a vacuum by electron beam welding. It was then subjected to HIP at 1150 °C and 150 MPa for 4 h, yielding HIP billets with dimensions of Φ (diameter) 150 mm × 152 mm, referred to as the As-HIP state. Those HIP billets were further processed through HEX and IF to produce forged blanks with dimensions of Φ180 mm × 53 mm, designated as the IF state. The extrusion parameters included a temperature of 1100 °C, a speed of 30mm/s, and an extrusion ratio of 4.7. Forging was executed in a two-stage process, with each heat maintained at 1080 °C, a speed of 0.005 s^−1^, and a total reduction of 55%. Samples, measuring Φ15 mm × 100 mm, were extracted from both As-HIP and IF state billets for HT in a Titan (H2) vacuum furnace (Ipsen, Souderton, PA, USA). The solution HT was performed at 1185 °C for 4 h with a cooling rate of approximately 150 °C/min, followed by aging HT at 815 °C for 8 h with a cooling rate of approximately 100 °C/min, designated as the As-HIP-HT and IF-HT, respectively.

### 2.2. Performance Testing

High-temperature tensile and creep tests were conducted for As-HIP-HT and IF-HT specimens. The tensile tests were performed on an ETM105D testing machine (Wance, Shenzhen, China) with cylindrical geometry specimens and a surface finish of 0.4 μm. The testing temperatures were set at 704 °C and 760 °C. During the test, an extensometer was placed on the tensile specimen to measure the elongation rate, with three test bars tested at each temperature. The creep tests were carried out on an RJ-50 creep testing machine (Kexin, Changchun, China). The creep test conditions were 800 °C/330 MPa, with three test bars tested for each condition.

### 2.3. Microstructure Observation

The microstructure of samples in four states, As-HIP, IF, As-HIP-HT, and IF-HT, was studied following grinding, polishing, and etching. Using a Quantax eFlash HR Electron Back-Scatter Diffraction (EBSD) (Bruker, Berlin, Germany) and a Sigma300 field emission Scanning Electron Microscope (SEM) (Carl Zeiss, Cambridge, UK), the grain size, γ′ phase, carbide morphology, and grain orientation spread (GOS) were observed. The composition of the micro-area was analyzed using an XFlash 6I60 129 eV Energy Dispersive Spectrometer (DES) (Bruker, Berlin, Germany). Carbide analysis was conducted using a Tecnai F20 Transmission Electron Microscope (TEM) (FEI, Hillsboro, OR, USA).

The fracture surface morphology was observed with a Nikon SMZ1270 Optical Microscope (OM) (Nikon, Tokyo, Japan) and SEM. The microstructure and dislocation configurations in the longitudinal section near the creep fracture surface were examined using SEM and TEM. Kalling’s etchant was used for microstructure and carbide observation, while γ′ precipitates were observed using a mixed etchant solution of 30% HNO_3_ +30% CH_3_COOH + 30% H_2_O + 10%HF. TEM samples were prepared by twin-jet thinning in a solution of 10% HClO_4_ and 90% CH_3_CH_2_OH-25 °C, utilizing Tenupol-5 equipment (Struers, Ballerup, Denmark).

## 3. Results and Discussion

### 3.1. Microstructure

#### 3.1.1. Grain Size

**Figure 1:**
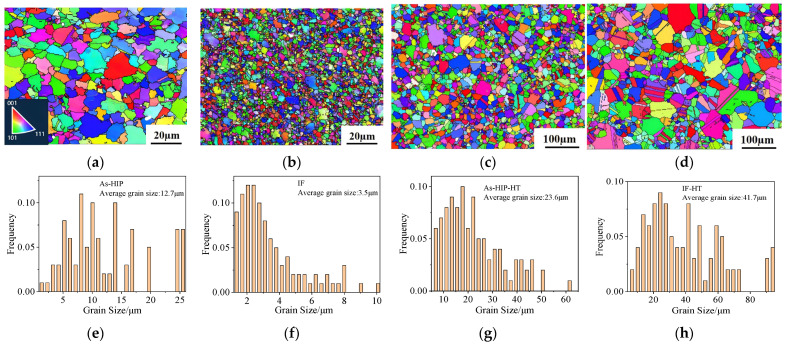
IPF maps of the FGH4113A alloy at different states: As-HIP (**a**); IF (**b**); As-HIP-HT (**c**); IF-HT (**d**) (the different colors in the IPF map represent the different orientations of the grains, as indicated in the lower left corner of (**a**)); grain size distribution at different states: As-HIP (**e**); IF (**f**); As-HIP-HT (**g**); IF-HT (**h**).

The grain structures of the FGH4113A alloy in four states, As-HIP, IF, As-HIP-HT, and IF-HT, were analyzed using EBSD to obtain inverse pole figure (IPF) maps. The grain size distributions were statistically evaluated (Figure 1). In the As-HIP alloy, dense equiaxed grains without distinct orientation were observed, with some partial twins visible in the matrix, indicating effective densification and deformation due to uniform three-directional compressive stress under the 1150 °C/4 h HIP process (Figure 1a). The IF alloy features equiaxed grains without significant texture distribution and features fine grains (Figure 1b). The average grain size of the As-HIP alloy was measured at 12.7 μm (ASTM 9.3), with a distribution range of from 1.5 to 25.5 μm (Figure 1e). Conversely, the grain size of the IF alloy was predominantly between 1.3 and 4.5 μm, averaging 3.5 μm (ASTM 13.0), and was approximately three ASTM grades finer than the As-HIP alloy (Figure 1e,f). The extrusion conducted at 1100 °C, a speed of 30 mm/s, and an extrusion ratio of 4.7 induces complete dynamic recrystallization (DRX) in the FGH4113A alloy, thereby refining the grain structure and establishing a favorable microstructure for isothermal forging [20]. The subsequent two-pass forging process with a forging temperature of 1080 °C, a forging speed of 0.005 s⁻^1^, and a total deformation reduction of 55%, further refines grains. The fine extruded grains in the FGH4113A alloy, under this forging process, are prone to discontinuous dynamic recrystallization (DDRX), a crucial mechanism of grain refinement observed during the hot deformation of alloys with medium to low stacking fault energy. This process involves nucleation and growth, promoting uniform grain refinement [21,22,23].

After HT, the grain size of the As-HIP-HT alloy displays a uniform distribution, with an average grain size of 23.6 μm (ASTM 7.5). Some annealed twins are visible within the grains (Figure 1c,g). The IF-HT alloy, on the other hand, exhibits equiaxed grains with no apparent orientation, a significant number of annealing twins, and a larger average grain size of 41.7 μm (ASTM 5.9) (Figure 1d,h). Comparing the microstructures, the IF-HT alloy displays larger grain sizes than the As-HIP-HT alloy after the same heat treatment. During sup-solvus HT, recrystallization occurs through strain-induced grain boundary migration, with the stored energy within the subgrains differing due to various dislocation densities [24]. After HEX and IF, the alloy contains additional stored energy, leading to more significant recrystallization and facilitating grain growth. Liu et al. [25] revealed that, when treatment temperatures surpassed the solution gamma prime solvus temperature, rapid grain growth occurred in FGH96 alloy disk blanks. Furthermore, the finer the initial grains, the higher the interface energy per unit area, which results in a higher growth rate. Given that the grains in the IF state are three ASTM grades finer than those in the As-HIP state alloy, they are more prone to coarsening during HT.

**Figure 2:**
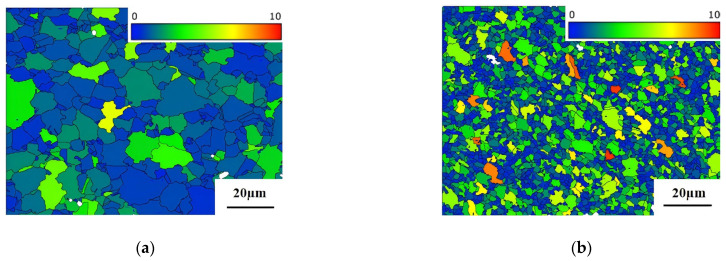

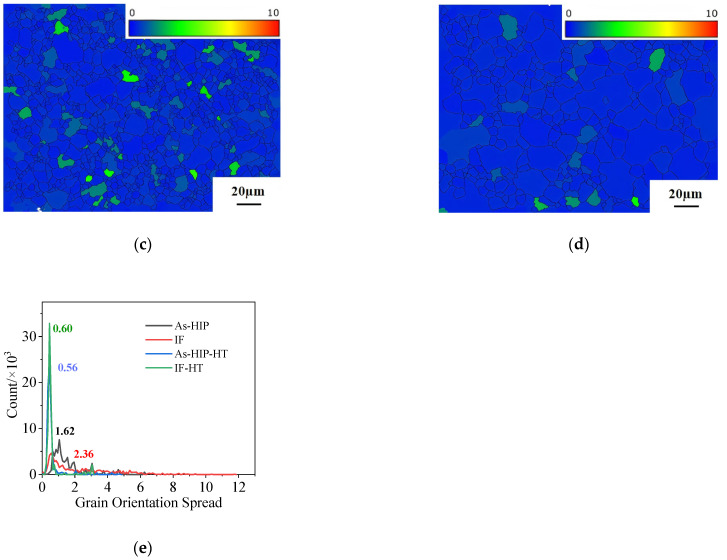
GOS maps of FGH4113A alloy in different states: As-HIP (**a**); IF (**b**); As-HIP-HT (**c**); IF-HT (**d**) (In the GOS map, the color indicates the degree of lattice distortion.); GOS distribution diagram (**e**).

During plastic deformation, energy is stored in the form of dislocations within the microstructure, providing the driving force for the nucleation and growth of recrystallized grains during subsequent annealing. This stored energy is an essential feature for optimizing microstructure and improving mechanical properties [26]. Figure 2 displays the grain orientation spread (GOS) maps and distribution of the alloys in various states. A higher GOS indicates severe lattice distortion and a higher dislocation density, while grains with a lower GOS exhibit a uniform strain distribution. The As-HIP and IF alloys exhibit larger GOS values, whereas the As-HIP-HT and IF-HT states demonstrate lower GOS values (Figure 2a–d). The GOS of As-HIP and IF alloys is distributed with mean values of 1.62° and 3.62° (Figure 2e), respectively, with the IF state exhibiting more dislocations and higher stored energy. After HT, the GOS of As-HIP-HT and IF-HT alloys decreases to 0.56° and 0.60° (Figure 2e), respectively. Due to the release of more stored energy during the HT process, the grains of IF-HT alloys are more prone to grow.

#### 3.1.2. γ′ Phase

**Figure 3:**
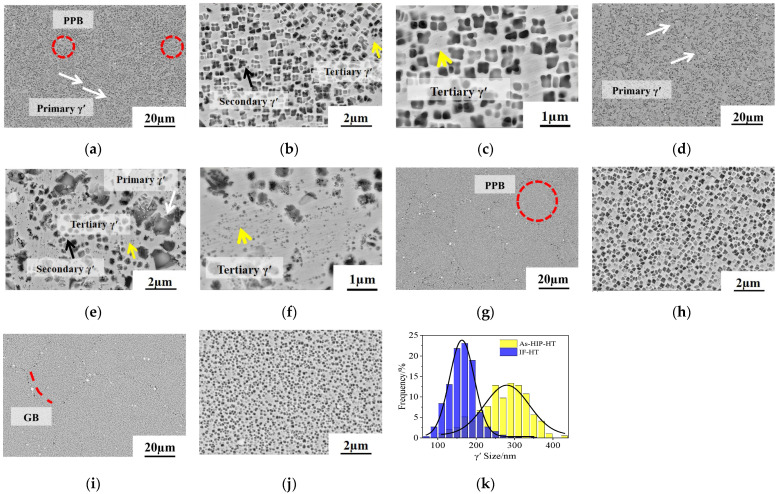
SEM photos of γ′ of the FGH4113A alloy in different states: As-HIP (**a**–**c**); IF (**d**–**f**); As-HIP-HT (**g**,**h**); IF-HT (**i**,**j**); secondary γ′ size distribution at As-HIP-HT and IF-HT states (**k**).

The γ′ phase morphologies in the alloy under various conditions are shown in Figure 3. In the As-HIP state, the alloy exhibits many coarse residual primary γ′ precipitates of varying sizes in the range of 1~4 μm, along with numerous butterfly-shaped secondary γ′ precipitates within the grains, surrounded by a few tertiary γ′ precipitates (less than 100 nm in size) (Figure 3a–c). In the IF alloy, a variety of coarse and irregular primary γ′ precipitates, measuring from 1 to 3 μm, are also present (Figure 3d). The deformation stored energy during forging enhances the diffusion of alloy elements, promoting the growth of large primary γ′ precipitates. Concurrently, recrystallization in the matrix further coarsens the γ′ phase, ultimately leading to the larger size of the primary γ′ [27]. The HIP process involves furnace cooling, allowing for sufficient time for the diffusion of solute atoms (e.g., Al and Ti). When those solute atoms reach saturation, secondary γ′ precipitates form. In the IF alloy, rapid cooling limits the timeframe for secondary γ′ precipitation, resulting in a lower content of fewer precipitates that appear fine and nearly spherical, while being surrounded by a tertiary γ′ smaller than 100 nm in size (Figure 3f). A limited number of PPBs are noted in the As-HIP alloy (red circles in Figure 3a), with large primary γ′ precipitates (from 1 to 2 μm) nearby. In contrast, PPBs are completely eliminated in the IF alloy (Figure 3d). Generally, the presence of PPBs can compromise the impact and creep properties of powder metallurgy superalloys [28].

After HT, the coarse primary γ′ precipitates at the grain boundaries in both the As-HIP-HT and IF-HT states are completely dissolved (Figure 3g,i). A small number of PPBs are visible in the As-HIP-HT alloy (red circle in Figure 3g). Fine and uniform secondary γ′ precipitates form within the grains of both alloys (Figure 3h,j). Figure 3k illustrates the size distribution of the secondary γ′ in the As-HIP-HT and IF-HT states, revealing a normal distribution pattern with mean values of 263 nm and 135 nm, respectively. Although the size difference is substantial, the area fractions of γ′ in both states remain relatively similar at 42.4% and 42.7%, respectively. The γ′ solvus temperature of FGH4113A is approximately 1158 °C [9]. Sup-solvus HT at 1185 °C dissolves the coarse primary γ′ at the grain boundaries, eliminating their pinning effect on the γ′ phase and accelerating the slip and climb rates at these boundaries, which, in turn, leads to a significant increase in grain size. The cooling rate is a pivotal factor influencing precipitate size [29,30]. Since both the As-HIP and IF alloys underwent identical HT processes in this study, the notable difference in the size of secondary γ′ precipitates can be attributed to other variables, as is further analyzed.

**Figure 4:**
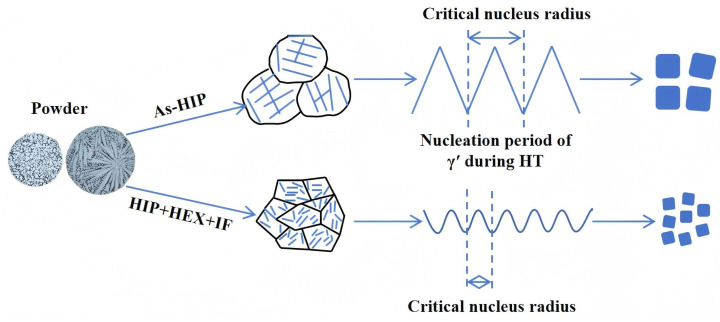
Schematic diagram for the nucleation and precipitation of secondary γ′ during HT.

Figure 4 illustrates the nucleation and precipitation of the secondary γ′ phase during HT for both processes. The internal microstructure of argon-atomized powder particles typically exhibits a dendritic or cellular morphology, which is primarily determined by the ratio of the temperature gradient at the solid–liquid interface front to the growth rate, as well as the cooling rate. Significant elemental segregation is observed, with elements such as Nb, Ti, Zr, and Al tending to accumulate in the interdendritic regions [31,32]. After HIP, the powder becomes fully dense on a macro scale. However, due to the different interdiffusion rates of stable elements in the γ phase between the matrix and γ′ phase, element segregation still exists in its substructure [33]. Such segregation cannot be fully eliminated during HT. During cooling, the γ′ phase nucleate is driven by compositional supercooling, with its critical nucleation radius determined by the segregation extent. After HEX + IF, the non-uniform substructures in the As-HIP state undergo thorough diffusion and fragmentation under elevated temperatures and stress, significantly alleviating compositional segregation, which results in a more uniform substructure. This homogeneity lowers the critical nucleation radius for the γ′ phase during the cooling process, promoting higher precipitation rates of finer secondary γ′ in the IF-HT state compared to the As-HIP-HT alloy.

#### 3.1.3. Carbides

**Figure 5:**
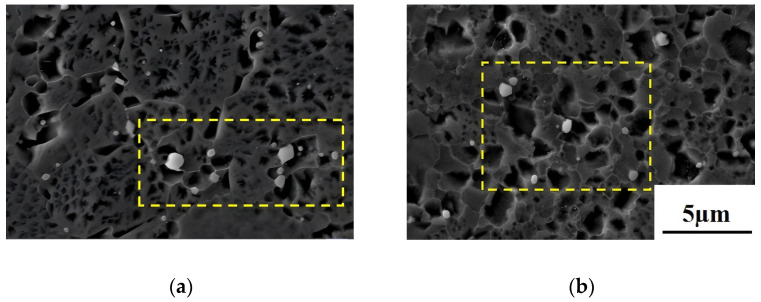

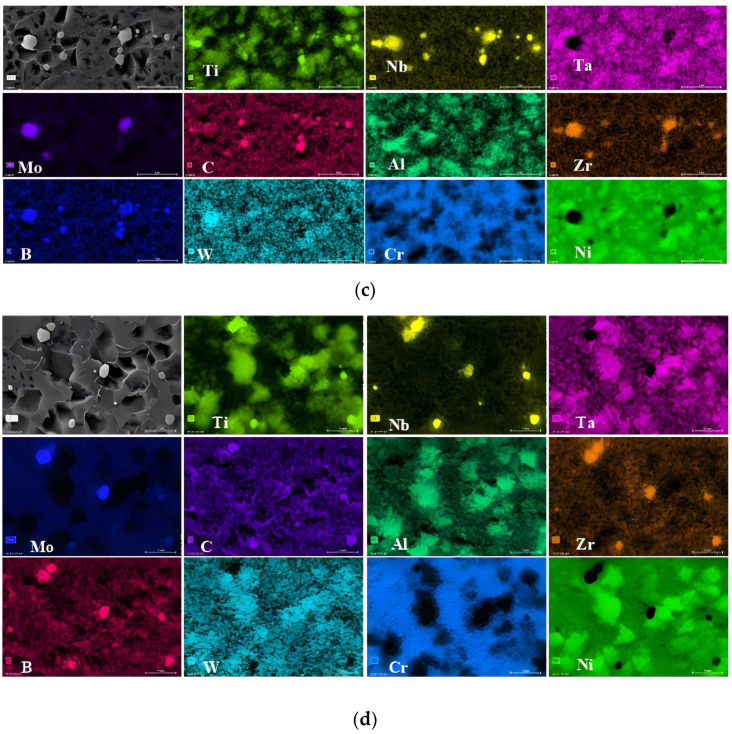
SEM photos of the carbide of the FGH4113A alloy at As-HIP (**a**); IF (**b**); EDS maps of the area in (**a**,**c,b**,**d**) (yellow rectangular frame).

The morphology, distribution, type, and quantity of carbides significantly influence the alloy’s mechanical properties. Figure 5 shows the morphology and EDS analysis of carbides in the alloy prepared via two processes. Both the As-HIP and IF alloys display blocky carbides, primarily concentrated at grain boundaries. The large carbides measure approximately 1.5 μm, while smaller carbides range from 0.1 μm to 0.5 μm (Figure 5a,b). EDS scanning reveals two predominant types of blocky carbides: MC carbides primarily composed of strong carbon-forming elements, such as Ti, Nb, Zr, and Ta, and M_6_C carbides, primarily composed of weak carbon-forming elements like W and Mo. Both types of carbides exhibit significant enrichment of B element; M_3_B_2_ or M_5_B_3_ type borides may also be present (Figure 5c,d).

**Figure 6:**
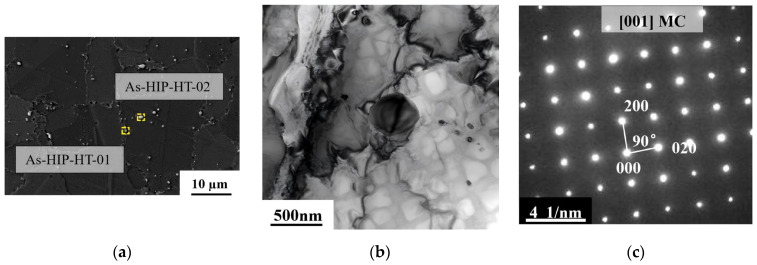

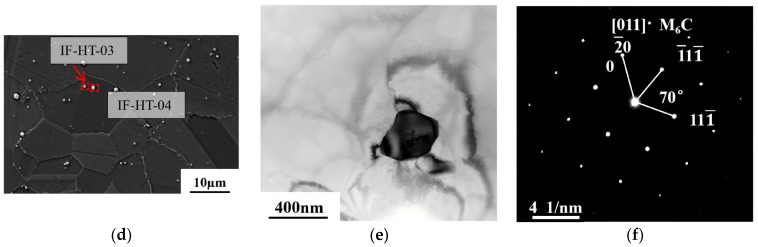
SEM photos for carbide of FGH4113A alloy at As-HIP-HT (**a**); IF-HT (**d**); TEM photos for granular carbide of IF-HT MC (**b**); M_6_C (**e**); HRTEM and selected area electron diffraction (SAED) pattern (inset) of the area in (**b,c,e,f**).

Figure 6 illustrates the carbide morphology and corresponding diffraction spots of the FGH4113A alloy after HT. After HT, initially clustered carbides at the grain boundaries disperse and distribute within both the grain boundaries and grains. These carbides appear as granular particles (Figure 6a,d). Figure 6b,c, as well as Figure 6e,f, depicts two types of granular carbides, MC and M_6_C, respectively, within the IF-HT state. Table 1 presents the composition of the granular precipitated carbides outlined in Figure 6a,d, which is consistent with the deformed state. After HT, the carbides in the alloy are still in the form of MC and M_6_C.

### 3.2. Tensile Properties

**Figure 7:**
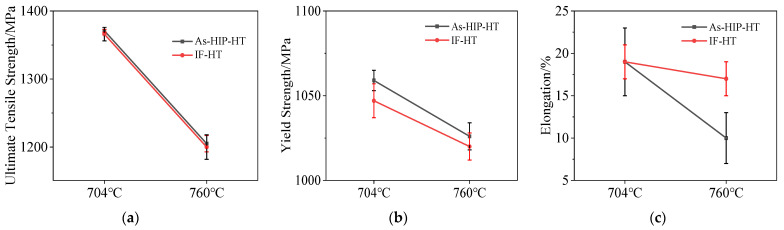
Tensile properties of FGH4113A alloy: ultimate tensile strength (**a**); yield strength (**b**); elongation (**c**).

The tensile properties of both As-HIP-HT and IF-HT FGH4113A were assessed at temperatures of 704 °C and 760 °C (Figure 7), with three specimens tested at each temperature. With the testing temperature increasing, the ultimate tensile strength (UTS), yield strength (YS), and elongation decrease significantly for both alloys. At both testing temperatures, the UTS of the As-HIP-HT and IF-HT alloys are not significantly different. Specifically, they are recorded at 1371 ± 2 MPa and 1366 ± 10 MPa at 704 °C, and 1205 ± 12 MPa and 1200 ± 18 MPa at 760 °C, respectively (Figure 7a). However, the YSs of the As-HIP-HT alloy at both 704 °C and 760 °C are higher than those in the IF-HT state, with an average increase of 33 MPa and 27 MPa, respectively (Figure 7b). At 704 °C, the elongation for both alloys is 19 ± 2%. At 760 °C, it drops to 17 ± 2% for the IF-HT alloy, but to 10% for the As-HIP-HT alloy (Figure 7c).

**Figure 8:**
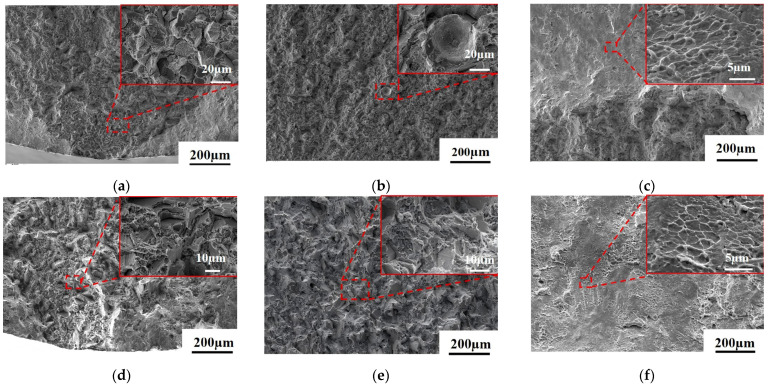
The tensile fracture morphology of the FGH4113A alloy at 704 °C As-HIP-HT (**a**–**c**); IF-HT (**d**–**f**); the crack initiation region (**a**,**d**); the propagation region (**b**,**e**); the transient fracture region (**c,f**).

Figure 8 illustrates the tensile fracture morphology of the As-HIP-HT and IF-HT alloys at 704 °C. The crack initiation region of the As-HIP-HT alloy exhibits quasi-cleavage, a mixed mode of transgranular and intergranular fracture (Figure 8a). The propagation region features both cleavage planes and ductile dimples, with prominent morphological characteristics along the PPB cracking (Figure 8b). In the transient fracture region, numerous elongated ductile dimples are visible (Figure 8c). In contrast, the crack initiation and propagation regions of the IF-HT alloy exhibit a mixed morphology of quasi-cleavage planes and ductile dimples, with no PPB fracture observed (Figure 8d,e). The transient fracture region of the IF-HT alloy has a smooth surface with elongated dimples, similar to the As-HIP-HT alloy (Figure 8f).

**Figure 9:**
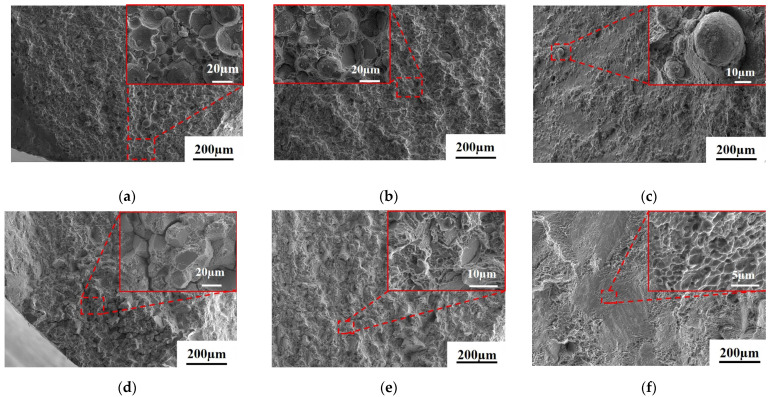
The tensile fracture morphology of FGH4113A alloy at 760 °C As-HIP-HT (**a**–**c**); IF-HT (**d**–**f**); the crack initiation region (**a**,**d**); the propagation region (**b**,**e**); the transient fracture region (**c**,**f**).

Figure 9 illustrates the tensile fracture morphology of the As-HIP-HT and IF-HT alloys at 760 °C. At this temperature, cracks in the As-HIP-HT alloy primarily initiate along the PPBs (Figure 9a). The propagation region displays a mixed transgranular and intergranular fracture pattern, with numerous PPB fracture features (Figure 9b). The relatively flat surface of the transient fracture region reveals ductile dimples, accompanied by numerous fractures along the PPBs (Figure 9c). The crack initiation region of the IF-HT alloy primarily exhibits intergranular fracture with secondary cracks (Figure 9d). The propagation region displays a mixed morphology of cleavage-like planes and ductile dimples (Figure 9e). The transient fracture region is smooth, revealing numerous elongated ductile dimples (Figure 9f).

When comparing the tensile fracture morphology characteristics of alloys in two states, only obvious PPB fracture characteristics are observed in the propagation region of the As-HIP-HT alloy fracture at 704 °C. However, at 760 °C, PPB fracture characteristics appear in all fracture areas, including the crack source region, propagation region, and transient fracture region. Under stress, cracks are more likely to initiate and propagate along the coarse carbides and γ′ phases surrounding the PPBs at 760 °C. In contrast, PPBs are completely eliminated in the IF-HT alloy, and the fracture morphology is significantly different from that of the As-HIP-HT alloy. Numerous studies have demonstrated that severe PPBs can hinder the diffusion and metallurgical bonding between particles, increasing the risk of crack initiation and negatively impacting mechanical properties [28,34]. At 704 °C and 760 °C, compared with the IF-HT alloy, the UTS of the As-HIP-HT alloy is comparable, but the YS is higher (Figure 7). This indicates that PPBs do not affect the high-temperature strength of the alloys, but reduce their plasticity to some extent. Similar findings were reported by Xia et al. [35], who discovered in their research that the presence of level 2 PPBs in alloys had minimal impact on high-temperature strength but it did affect elongation and other properties to some degree, which is consistent with the results of this study.

The tensile properties of alloys are closely related to their microstructures. According to the Hall–Petch relationship, grain boundaries act as strong barriers to dislocation movement, and fine grains provide more such barriers. Therefore, finer grains generally result in higher strength [36]. As previously mentioned, the average grain sizes of the As-HIP-HT and IF-HT FGH4113A alloys are 23.1 μm and 31.7 μm, respectively. Consequently, the As-HIP-HT alloy, which has finer grains, exhibits a higher YS than the IF-HT alloy.

### 3.3. Creep Properties

**Figure 10:**
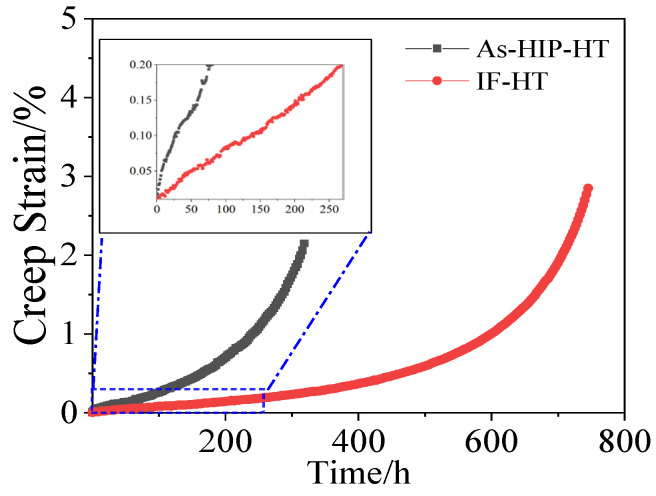
The creep curve of the FGH4113A alloy at 800 °C/330MPa.

Figure 10 displays the creep curves of the As-HIP-HT and IF-HT alloys tested at 800 °C/330 MPa. Under such conditions, neither alloy exhibits a distinct deceleration creep stage. The curves primarily consist of the secondary and tertiary deformation, with the IF-HT state demonstrating a longer steady-state creep stage compared to the As-HIP-HT alloy. The creep life of the As-HIP-HT alloy is 319 h, with a total creep deformation of approximately 2% (Figure 10). In contrast, the creep life of the IF-HT alloy is 782 h, with a total creep strain of approximately 3%. The creep life of the IF-HT alloy is extended by approximately 145% compared to that of the As-HIP-HT alloy. Three samples of each alloy were tested, and the average creep times corresponding to 0.2% residual creep were found to be 86 h and 205 h, respectively (enlarged view in Figure 10).

**Figure 11:**
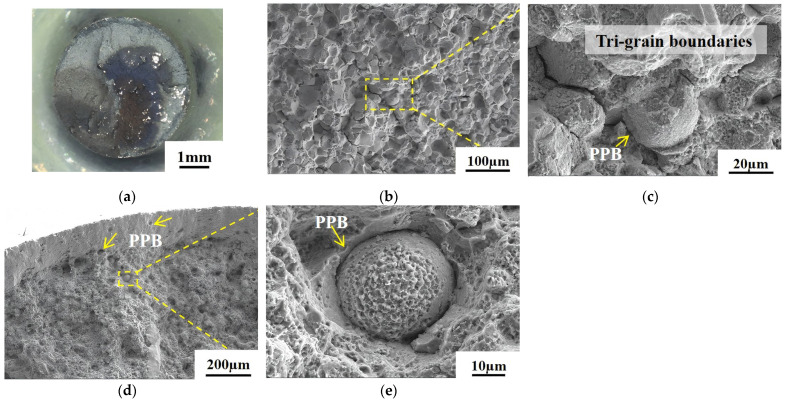
The creep fracture morphology of the FGH4113A alloy at the As-HIP-HT macroscopic fracture (**a**); the propagation region (**b**,**c**); the transient fracture region (**d**,**e**).

**Figure 12:**
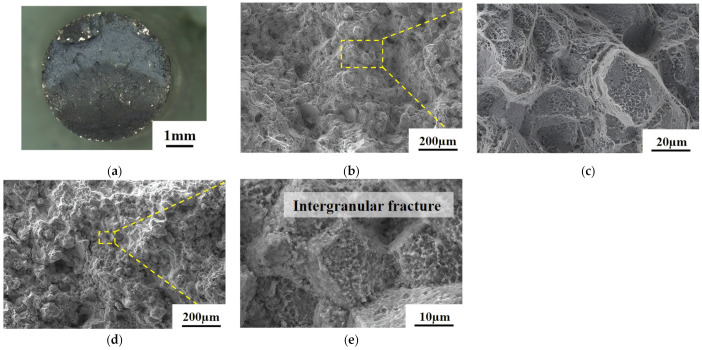
The creep fracture morphology of the FGH4113A alloy at the IF-HT macroscopic fracture (**a**); the propagation region (**b**,**c**); the transient fracture region (**d**,**e**).

Figure 11 and Figure 12 depict the creep fracture morphology of the As-HIP-HT and IF-HT alloys at 800 °C/330 MPa, with respective creep lives of 319 h and 782 h. Figure 11a illustrates the macroscopic morphology of the creep fracture in the As-HIP-HT alloy. The creep fracture surface appears relatively flat with no evident necking, and the crack initiation region is located at the edge. The shear lip area is small, while the propagation region covers a larger area and appears blue–gray in color. The crack source region morphology is shown in Figure 11b, revealing that the primary fracture is an intergranular fracture. Since the grain boundary serves as the origin of cracks, creep cavities are predominantly formed on the grain boundary under vertical tensile stress. Under higher loads, cracks tend to initiate at the tri-grain boundaries (red arrow in Figure 11c). Meanwhile, in the crack initiation region, morphological features of the cracking along the PPBs are visible (yellow arrow in Figure 11c). PPB holes are visible both in the stable expansion region and in the smaller transient fracture region (arrows in Figure 11d). Deep fine dimples are visible on the surface of the PPB powder particles (arrow Figure 11e).

**Figure 13:**
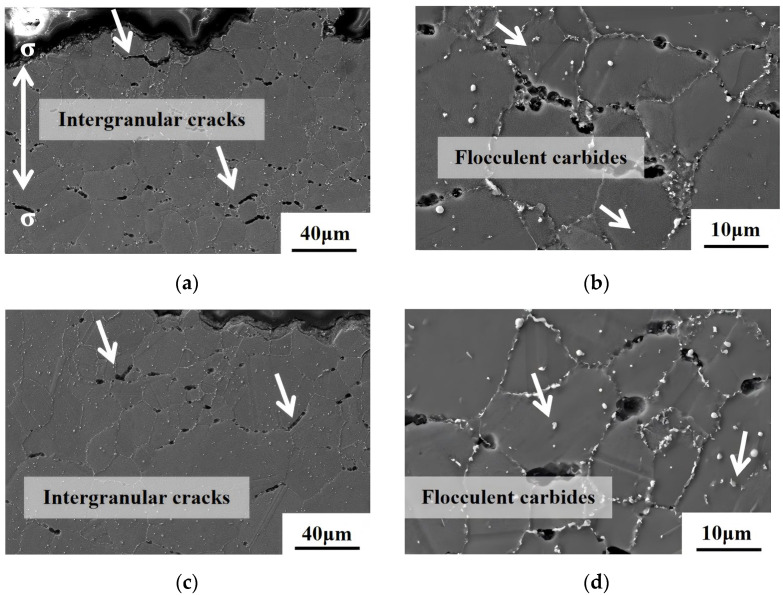
SEM photos of longitudinal sectional morphology of the creep fracture of the FGH4113A alloy As-HIP-HT (**a**,**b**); IF-HT (**c**,**d**).

Figure 13 illustrates the longitudinal sectional microstructure morphology of the creep fracture surfaces of the As-HIP-HT and IF-HT alloys tested at 800 °C/330 MPa. Both alloys exhibit numerous intergranular cracks near the fracture. At 800 °C, the isothermal strength of the alloy is exceeded, indicating that the grain boundary strength is weaker than the grain strength. The crack propagation direction is perpendicular to the stress axis (double arrow in Figure 13). During creep deformation, grain boundary voids accumulate, and microcracks propagate until creep rupture occurs (Figure 13a,c). After prolonged exposure to high temperature and high stress, carbides are observed to be enriched at grain boundaries in both alloys, with a small amount of flocculent carbides precipitating within the grains (Figure 13b,d).

### 3.4. TEM Analysis of Microstructure near the Creep Fractures

**Figure 14:**
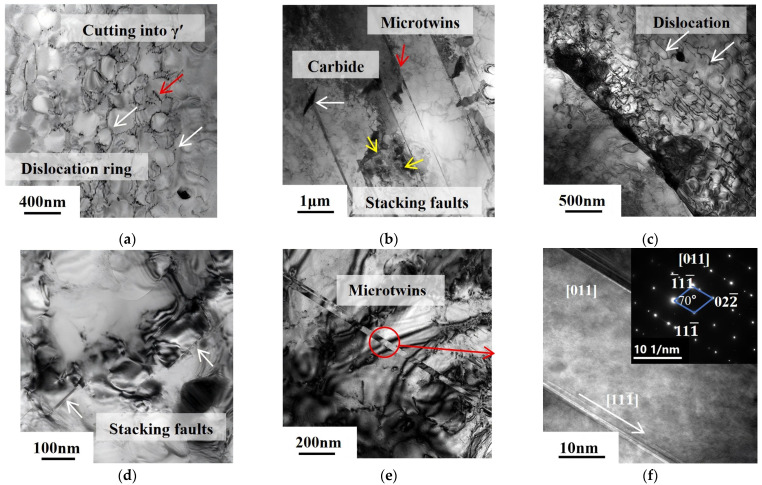
TEM photos of the creep fracture of the FGH4113A alloy As-HIP-HT (**a**,**b**); IF-HT (**c**–**f**).

Figure 14 illustrates the dislocation configuration at the creep fracture of the FGH4113A alloy in the As-HIP-HT and IF-HT states. After the creep fracture of the As-HIP-HT alloy, the secondary γ′ transformed from a rounded cubic to a near-spherical shape, indicating element diffusion within the alloy during creep. A significant number of dislocation loops are observed in the matrix, resulting from dislocation lines bowing out between the γ′ phases, orbiting around the γ′ phase, and intersecting, ultimately forming a dislocation network primarily composed of dislocation loops (white arrows in Figure 14a). During creep, moving dislocations in the matrix encounter a network of dislocations, triggering a reaction that alters the direction of dislocation motion. This prompts the dislocations to climb into the γ′ phase, resulting in only a small number of dislocations cutting into the large γ′ phase [37] (red arrow in Figure 14a). Additionally, the alloy contains partial stacking faults and microtwins (yellow and red arrows in Figure 14b). Due to the dislocations sheared into the γ′ phase possessing varying Burgers vectors, they exhibit a distinct morphology. Specifically, dislocations inserted into the γ′ phase undergo decomposition, resulting in a configuration of incomplete dislocations accompanied by stacking faults. In the nickel-based alloy matrix, when the a/2 {110} dislocations slip and encounter the γ′ phase, they split into two incomplete dislocations: a/6 {112} and a/3 {11$\bar{2}$}. Upon entering the γ′ phase, the a/6 {112} dislocation gives rise to complex stacking faults with elevated energy. To minimize the energy, the a/6 {112} dislocations and extrinsic stacking faults emerge on the {111} plane [38,39]. When the stacking fault atomic layer exceeds three layers, pseudo microtwins form, and microtwins emerge at the stacking fault interface after atomic rearrangement [40]. As is evident from Figure 14a,b, the creep deformation mechanisms of the As-HIP-HT alloy primarily involve Orawan dislocation loops and deformation twins.

After creep fracture occurs in the IF-HT alloy, numerous deformed dislocations cut through the γ′ phase in the matrix, demonstrating a typical mechanism of dislocation cutting through the γ′ phase (arrows in Figure 14c). As the creep progresses to later stages, the strain continuously increases, leading to a significant accumulation of deformation dislocations at the interface. When the stress concentration resulting from dislocation pile-up increases, some of the γ′ phase interface dislocation networks are disrupted. When stress concentration occurs again, it allows the deformation dislocations to cut into the γ′ phase. When superdislocations penetrate into the γ′ phase, decomposition occurs, leading to the formation of stacking faults (arrows in Figure 14d). Additionally, the matrix contains partial microtwins, which arise from dislocations sliding in [2$\bar{1}1$] direction within adjacent (11$\bar{1}$) planes [41] (Figure 14e,f). Analysis reveals that the primary creep deformation mechanisms of the IF-HT alloy involve dislocations cutting through the γ′ phase and dislocation sliding, resulting in the formation of microtwins.

Under prolonged exposure to high temperatures and stress during creep, flocculent carbides precipitated within the grains of the two alloys, hindering the further expansion of microtwins during the creep process (white arrow in Figure 14b). Liu et al. [42], while investigating the precipitation behavior of carbides during creep and their impact on creep properties, discovered that two types of carbides, M_23_C_6_ and M_6_C, precipitated during creep. The dispersed carbide particles impeded dislocation movement through dislocation entanglement and pinning, thereby enhancing creep properties, similar to the findings in this study.

The creep behavior of powder nickel-based superalloys is primarily influenced by the γ′ phase, grain boundary characteristics, test temperature, and stress. Peng et al. [43] investigated the mechanism by which the γ′ phase affects creep properties in a FGH96 powder alloy. They discovered that, as the γ′ phase increases, its hindrance to dislocation slip diminishes, resulting in a lower creep rate and longer creep life. The size of the secondary γ′ phase in the As-HIP-HT alloy is 263 nm, which is relatively large. In this alloy, a significant number of dislocations surround the γ′ phase as Orawan dislocations. Conversely, the size of the secondary γ′ in the IF-HT alloy is 135 nm, which is smaller. Here, dislocations predominantly move by cutting through the γ′ phase, which demands greater stress. By comparing the stacking fault morphology of the alloy in two states, it is evident that the dislocations exhibit stronger movement capabilities, enabling the simultaneous activation of slip systems in different directions within the alloy. The greater the number of stacking fault directions, the faster the creep rate becomes. Referring to Figure 14b,d, which depicts the stacking faults in two different states, it is clear that the As-HIP-HT state forms a greater number of stacking faults in various directions, leading to a relatively higher creep rate.

## 4. Conclusions

The FGH4113A alloy was processed using two different thermal–mechanical processes, and the impact of the processes on the alloy’s microstructure and mechanical properties was compared; based on this, the following conclusions can be drawn:

(1) Both the As-HIP and IF alloys underwent sufficient recrystallization, with average grain sizes of 12.7 μm (As-HIP) and 3.5 μm (IF). The As-HIP alloy exhibited distinct PPBs. The grain sizes significantly increased after HT for both the As-HIP-HT and IF-HT alloys. Due to higher storage energy, the grain size of the IF-HT state is larger than that of the As-HIP-HT alloy.
(2) In both the As-HIP-HT and IF-HT alloys, the primary γ′ precipitates completely dissolved, while significant differences in secondary γ′ were observed due to compositional non-uniformity in the substructure of the As-HIP state, resulting in a relatively long period for γ′ phase nucleation, leading to fewer, larger γ′ particles compared to the more uniform substructure of the IF state, which allowed for more numerous, smaller γ′ precipitates. Both states exhibited similar dispersed MC and M_6_C carbides pre- and post-HT.
(3) At both 704 °C and 760 °C, the YS of the As-HIP-HT state was higher than that of the IF-HT alloy. The plasticity of the As-HIP-HT state alloy decreased sharply from 19% to 10% as the temperature rose, while the plasticity of the IF-HT state remained relatively unchanged. The presence of PPBs in the As-HIP-HT state had minimal impact on the alloy’s strength, but it did reduce the alloy’s plasticity to some extent.
(4) Creep testing at 800 °C/330 MPa revealed a significant difference in creep life between the As-HIP-HT and IF-HT alloys. The IF-HT alloy exhibited lower creep rates and longer lifespans owing to its finer γ′ precipitate size. In the As-HIP-HT state, the primary mechanisms of creep deformation were Orawan dislocation loops and deformation twins. Conversely, in the IF-HT state, the main mechanisms were dislocations cutting through the γ′ phase, dislocation slip, and microtwins.

The application scenarios of the as-HIP near-net-shaped FGH4113A alloy need to be evaluated for use in non-critical aviation components. However, the performance characteristics of the extruded and forged versions of FGH4113A point to significant potential for applications in critical components.

## Figures and Tables

**Table 1:** Carbide composition in the heat-treated alloy (wt.%).

Spectrum	Al	Ti	Cr	Co	Ni	Zr	Nb	Mo	Hf	Ta	W	Al + Ti + Ta + Nb	W + Mo
As-HIP-HT-01	0.4	3.6	17.6	7.4	15.3	0.8	2.9	26.0	0.1	1.6	24.3	8.5	50.4
As-HIP-HT-02	1.7	12.8	10.5	13.5	30.2	1.0	9.8	3.6	2.9	10.8	3.3	35.1	6.8
IF-HT-03	0.4	3.2	17.6	5.5	9.9	1.4	3.4	29.9	0.2	1.6	26.9	8.6	56.8
IF-HT-04	1.2	18.4	6.2	8.6	20.8	1.3	15.6	3.0	3.3	18.4	3.1	53.6	6.1

## Data Availability

The original contributions presented in this study are included in the article, further inquiries can be directed to the corresponding authors.
